# Supplementary material for: Large scale and regional demographic responses to climatic changes in Europe during the Final Palaeolithic
Source: PLoS One. 2025 Apr 2;20(4):e0310942. doi: 10.1371/journal.pone.0310942 (PMC11964466; doi:10.1371/journal.pone.0310942)
Supplement: S1 file — (DOCX) [file pone.0310942.s001.docx]

# **Information on Maps**

All maps were created using QGIS, version 3.34.7 “Prizren” in projection WGS 84, EPSG 3035.

The calculation of the palaeodemographic estimates, including the modelling of the optimally describing isoline / Core Areas (see [1] and https://github.com/C-C-A-A/CologneProtocol-MapInfo) and the convex hulls of the raw material catchment areas (RMCA), was conducted using MapInfo Professional 8.5, projection Lambert azimuthal Flächentreu - Baltikum (Bereich 90).

The GIS-sources used to create the basemap were taken from <https://www.naturalearthdata.com/>:

Relief shading and hypsography:

1:10m Gray Earth: Gray Earth with Shaded Relief and Hypsography

Coastline / Ocean:

1:50m Physical Vectors, Ocean

Rivers and Lakes

1:50m Rivers, Lake Centerlines

Additionally, we included for areas > 45° N:

Open access data from EPHA – European prehistoric and historic atlas (<https://zbsa.eu/european-prehistoric-and-historic-atlas/>):

- [Drainage Systems](https://zbsa.eu/drainage-systems/)
- [Allerød](https://zbsa.eu/allerod/)
- [Dryas III](https://zbsa.eu/younger-dryas/)

The GIS-created maps were reworked and a legend was added using Adobe Illustrator CS6.

# **References**

1. Schmidt I, Hilpert J, Kretschmer I, Peters R, Broich M, Schiesberg S, et al. Approaching prehistoric demography: proxies, scales and scope of the Cologne Protocol in European contexts. Phil Trans R Soc B. 2021;376: 20190714. doi:10.1098/rstb.2019.0714
