# Supplementary material for: Large scale and regional demographic responses to climatic changes in Europe during the Final Palaeolithic
Source: PLoS One. 2025 Apr 2;20(4):e0310942. doi: 10.1371/journal.pone.0310942 (PMC11964466; doi:10.1371/journal.pone.0310942)
Supplement: S1 Table — The table comprises only data that were considered for calculation in this study (n = 53). Below each region, the corresponding quartiles (Q1, Q2, Q3) used to calculate the palaeodemographic estimates are shown (see table 1). (DOCX) [file pone.0310942.s002.docx]

**S1 Table.** **Sites with data on Raw Material Catchment Areas (RMCA) assigned to GI-1d-a.**

| **Region** | **Site** | **RMCA in km^2^** |  | **Reference** |  |  |
| --- | --- | --- | --- | --- | --- | --- |
| NW |  |  |  |  |  |  |
|  | Bad Breisig | 549 |  | [1]; pers. comm. B. Gehlen |  |  |
|  | Eindegoorheide 1 | 630 |  | [2] |  |  |
|  | Geldrop 3-1 | 797 |  | [3] |  |  |
|  | Urbar (20) | 853 |  | [4] |  |  |
|  | Tongeren-Plinius | 2670 |  | [5] |  |  |
|  | Wesseling-Eichholz | 3665 |  | [6,7]; pers. comm. B. Gehlen |  |  |
|  | Horn-Haelen | 3909 |  | [8] |  |  |
|  | Gönnersdorf | 4959 |  | [9] |  |  |
|  | Heythuysen-De Fransman | 5117 |  | [8] |  |  |
|  | Kettig | 5220 |  | [10] |  |  |
|  | Andernach-Martinsberg | 5818 |  | [11]; pers. comm. B. Gehlen |  |  |
|  | Rüsselsheim 122 (A, B) | 5896 |  | [12] |  |  |
|  | Niederbieber II | 7284 |  | [11]; pers. comm. B. Gehlen |  |  |
|  | Niederbieber IV | 7284 |  | [11]; pers. comm. B. Gehlen |  |  |
|  | Ruien "Rosalinde" | 7562 |  | [13] |  |  |
|  | Kartstein | 9665 |  | [14]; pers. comm. B. Gehlen |  |  |
| *NW* | *Q1* | *2670* |  |  |  |  |
|  | *Q2* | *5117* |  |  |  |  |
|  | *Q3* | *5896* |  |  |  |  |
| NE |  |  |  |  |  |  |
|  | Całowanie | 605 |  | [15] |  |  |
|  | Jeskyně tří volů (Jeskyne tri volu) | 747 |  | [16] |  |  |
|  | Tarnowa 1 | 975 |  | [15] |  |  |
|  | Dzierżysław (Dzierzyslaw) | 1302 |  | [17] |  |  |
|  | Kvíc | 1767 |  | [18] |  |  |
|  | Kraków-Bieżanów (Krakow-Biezanow) | 2129 |  | [19] |  |  |
|  | Trebomyslice 1 | 2318 |  | [20] | |  |
|  | Hostim I - Krápníková jeskyne | 2729 |  | [16] |  |  |
|  | Uckersdorf | 24,016 |  | [21] |  |  |
|  | Wöllershof-Ketzerrang | 24,923 |  | [21] |  |  |
| *NE* | *Q1* | *1057* |  |  |  |  |
|  | *Q2* | *1948* |  |  |  |  |
|  | *Q3* | *2626* |  |  |  |  |
| SE |  |  |  |  |  |  |
|  | Abri de Douattes; Douattes Est | 689 |  | [22] |  |  |
|  | Sattenbeuren-Kieswerk | 876 |  | [23] |  |  |
|  | Gunzwil-Beromünster | 1523 |  | [22] |  |  |
| [SE] | Wauwil-Sandmatt 25 | 1882 |  | [24] |  |  |
|  | Birseck-Ermitage | 2352 |  | [22] |  |  |
|  | Abri Unter den Seewänden | 2857 |  | [25]; pers. comm. B. Gehlen |  |  |
|  | "Gemeindebeunden" Bad Buchau-Kappel | 3056 |  | [26] |  |  |
|  | Neumühle, Abri / Neumuhle | 3110 |  | [22] |  |  |
|  | Geispel | 3474 |  | [22] |  |  |
|  | Monruz (Neuchâtel) | 3550 |  | [22]; pers. comm. J. Affolter / B. Gehlen |  |  |
|  | Fru, La (423) | 3864 |  | [27] |  |  |
|  | Champreveyres / Hauterive-Champréveyres | 5023 |  | [22] |  |  |
|  | Cure, Abri de la | 7467 |  | [22]; pers. comm. J. Affolter / B. Gehlen |  |  |
|  | Lüscherzmoos | 8297 |  | [22]; pers. comm. J. Affolter / B. Gehlen |  |  |
|  | Cham-Grindel III | 9026 |  | [22] |  |  |
|  | Langrüti | 13,833 |  | [22] |  |  |
|  | Fürsteiner | 15,239 |  | [22] |  |  |
|  | Schlössel 1 | 20,434 |  | pers. comm. J. Affolter / B. Gehlen |  |  |
|  | Hopferau-Pertlesbichl | 28,503 |  | [28]; pers. comm. B. Gehlen; |  |  |
| *SE* | *Q1* | *2604* |  |  |  |  |
|  | *Q2* | *3550* |  |  |  |  |
|  | *Q3* | *8662* |  |  |  |  |
| SW |  |  |  |  |  |  |
|  | Chaloignes, Les | 571 |  | [29] |  |  |
|  | Forcas I | 755 |  | [30] |  |  |
|  | Balzola, Cueva de | 2401 |  | [31] |  |  |
|  | Troubat, La Grotte-Abri de/ Moulin | 2522 |  | [32] |  |  |
|  | Rhodes II, L'Abri | 12,788 |  | [33] |  |  |
|  | Béraud, Grotte | 28,672 |  | [34] |  |  |
|  | Mas d'Azil | 40,446 |  | [35] |  |  |
| *SW* | *Q1* | *1578* |  |  |  |  |
|  | *Q2* | *2522* |  |  |  |  |
|  | *Q3* | *20,730* |  |  |  |  |
|  |  |  |  |  |  |  |

The table comprises only data that were considered for calculation in this study (n = 53). Below each region, the corresponding quartiles (Q1, Q2, Q3) used to calculate the palaeodemographic estimates are shown (see **table 1**).

**References**

1. Grimm SB. Ein spätallerödzeitlicher Fundplatz bei Bad Breisig, Kreis Ahrweiler. Trierer Zeitschrift. 2004;28: 11–32.

2. Verbeek C. Epipaleolithische en Mesolithische sites in het “Ruilverkavelingsblok Weelde” (prov. Antwerpen). Notae Praehistoricae. 1997;17: 81–84.

3. Deeben J. De laatpaleolithische en mesolithische sites bij Geldrop (N. Br.). Deel 5. Archeologie. 1999;9: 3–35.

4. Baales M, Mewis SU, Street M. Der Federmesser-Fundplatz Urbar bei Koblenz. Jahrbuch des Römisch-Germanischen Zentralmuseums Mainz. 1996;43: 241–79.

5. Dijkstra P, Bink M, De Bie M, Vynckier G, Van Rechem H, Dyselinck T. Laatpaleolithische vindplaatsen op het Plinius-terrein bij Tongeren (prov. Limburg). Notae Praehistoricae. 2006;26: 109–124.

6. Parow-Souchon H, Heinen M. Raw material economy and mobility in the Rhenish Allerød. Quartär. 2017;64: 157–177. doi:10.7485/QU64_7

7. Heinen M. Der Federmesser-Horizont am Niederrhein und im angrenzenden Mittelgebirgsraum: Regionale und interne Organisation. In: Baales M, Pasda C, editors. „All der holden Hügel ist keiner mir fremd .“: Festschrift zum 65 Geburtstag von Claus-Joachim Kind. Bonn: Rudolf Habelt; 2019.

8. Stoop D. Federmesser mobility patterns in the Western Meuse area, Limburg, the Netherlands: the case studies of Horn-Haelen and Heythuysen-de Fransman I. Leiden: Leiden University; 2014. Available: https://hdl.handle.net/1887/28507

9. Baales M. Archäologie des Eiszeitalters: frühe Menschen an Mittelrhein und Mosel. Koblenz: Gesellschaft für Archäologie an Mittelrhein und Mosel; 2005.

10. Baales M. Neue Untersuchungen zum Spätpaläolithikum des Neuwieder Beckens : einige Aspekte des Federmesser-Fundplatzes Kettig, Kr. Mayen-Koblenz. Den Bogen spannen: Festschrift für Bernhard Gramsch zum 65 Geburtstag. Weissbach: Beier & Beran; 1999. pp. 55–66.

11. Floss H. Rohmaterialversorgung im Paläolithikum des Mittelrheingebietes. Bonn: Habelt; 1994.

12. Loew S. Rüsselsheim 122 und die Federmessergruppen am Unteren Main. Ph.D., Universität zu Köln. 2006. Available: http://kups.ub.uni-koeln.de/id/eprint/2079

13. Crombé P, Sergant J, Verbrugge A, De Graeve A, Cherretté B, Mikkelsen J, et al. A sealed flint knapping site from the Younger Dryas in the Scheldt valley (Belgium): Bridging the gap in human occupation at the Pleistocene–Holocene transition in W Europe. J Archaeol Sci. 2014;50: 420–439. doi:10.1016/j.jas.2014.07.021

14. Baales M. Umwelt und Jagdökonomie der Ahrensburger Rentierjäger im Mittelgebirge. Bonn: Dr. Rudolf Habelt GmbH; 1996.

15. Sulgostowska Z. Final Palaeolithic Societies’ Mobility in Poland as Seen from the Distribution of Flints. Arch Baltica. 2006;7: 36–42.

16. Oliva M. Encyklopedie paleolitu a mezolitu českých zemí. Vydání 1. Brno: Moravské zemské muzeum; 2016.

17. Tarbska J, Walanus A, Ciesielczuk J, Samek L, Dutkiewicz E. Ferruginous Raw Material Sources for Palaeolithic in Poland (Central Europe)? Provenance Studies: Occurrence, Litostratigraphy and Application. Jerusalem; 2008. Available: https://www.ndt.net/?id=6175

18. Vencl S. Prehistory of Bohemia 1: The Palaeolithic and Mesolithic. Praha: Archeologický ústav AV CR; 2013.

19. Stefański D, Wilczyński J. Extralocal raw materials in the Swiderian Culture: Case study of Kraków-Bieżanów sites. Anthropologie. 2012;50: 427–442. doi:http://www.jstor.org/stable/26272422

20. Šída P, Pokorný P. Determining the archaeological potential of the landscape using Quaternary geological mapping in the Třebon region, south Bohemia. Arch Rozhledy. 2011;63: 485.

21. Sauer F. Late Palaeolithic Land Use Patterns in Bavaria. Ph.D., Friedrich-Alexander-Universität. 2018. Available: urn:nbn:de:bvb:29-opus4-92875

22. Affolter J. Silexrohstoffe - Schlüssel zu Analyse von Beziehungsnetzen. Die letzten Wildbeuter der Eiszeit - Neue Forschungen zum Spätpaläolithikum im Kanton Basel-Landschaft. Bern: Schwabe Verlag; 2015. pp. 198–209.

23. Kind C-J. Sattenbeuren - Kieswerk, ein spätpaläolithischer Uferrandlagerplatz am Federsee. Fundb Baden-Württemberg. 1995;20: 159–194.

24. Nielsen EH, Affolter J. Wauwil Station 25-Sandmatt: eine spätpaläolithische Fundstelle im Wauwilermoos. Luzern: Kantonaler Lehrmittelverlag; 1999.

25. Gehlen B. Rast am Fuße der Alpen: Die allerødzeitliche Abristation “Unter den Seewänden” bei Füssen im Ostallgäu. Zeit-Räume: Gedenkschrift für Wolfgang Taute. Propylaeum; 2001. pp. 475–552. doi:10.11588/PROPYLAEUM.245.327

26. Jochim MA, Kind C-J, Kleinmann A, Merkt J, Stephan E. Eine spätpaläolithische Fundstelle am Ufer des Federsees: Bad Buchau-Kappel, Flurstück Gemeindebeunden. Fundber Baden-Württemberg. 2015;35: 37–134. doi:10.11588/FBBW.2015.0.44521

27. Mevel L, Pion G, Fornage-Bontemps S. Changements techniques et géographie culturelle à l’extrême fin du Paléolithique dans les Alpes du nord françaises. Les stratigraphies de l’abri de La Fru (Savoie) revisitées. In: Jaubert J, Fourment N, Depaepe P, editors. Transitions, ruptures et continuité en préhistoire: actes du XXVII Congrès préhistorique de France: Bordeaux-Les Eyzies: 31 mai-5 juin 2010 Volume 1. Paris: Société Préhistorique Française; 2014. pp. 527–546.

28. Gehlen B. Steinzeitliche Funde im östlichen Allgäu. In: Küster H, editor. Vom Werden einer Kulturlandschaft Vegetationsgeschichtliche Studien am Auerberg (Südbayern). Weinheim; 1988. pp. 195–209.

29. Marchand G, Blanchet S, Chevalier G, Gallais JY, Le Goffic M, Naudinot N, et al. La fin du Tardiglaciaire sur le Massif armoricain : territoires et cultures matérielles. Paléo. 2004;16: 137–170.

30. Sánchez de la Torre M. Detecting human mobility in the Pyrenees through the analysis of chert tools during the Upper Palaeolithic. J Lithic Stud. 2014;1: 263–279.

31. García Rojas M. Dinámicas de talla y gestión de las materias primas silíceas a finales del pleistoceno en el País Vasco. Ph.D., Universidad del País Vasco. 2014. Available: http://hdl.handle.net/10810/18142

32. Barbaza M. Environmental changes and cultural dynamics along the northern slope of the Pyrenees during the Younger Dryas. Quat Int. 2011;242: 313–327. doi:10.1016/j.quaint.2011.03.012

33. Fat Cheung C, Chevallier A, Bonnet-Jacquement P, Langlais M, Ferrié J-G, Costamagno S, et al. Comparaison des séquences aziliennes entre Dordogne et Pyrénées: état des travaux en cours. Les groupes culturels de la transition Pléistocène - Holocène entre Atlantique et Adriatique: actes de la séance de la Société préhistorique française Bordeaux 24-25 mai 2012. Paris: Société préhistorique française; 2014. pp. 17–44.

34. Surmely F, Quinqueton A, Virmont J. Le gisement épipaléolithique ancien de la grotte Béraud à Saint-Privat-d’Allier (Haute-Loire, France). hal-00350923 , version 1. 2001.

35. Kegler JF. Das Azilien von Mas d’Azil. Der chronologische und kulturelle Kontext der Rückenspitzengruppen in Südwesteuropa. Mit einem Beitrag von Jan F. Kegler und Stefan R. Loew. Ph.D., Universität zu Köln. 2007. Available: http://kups.ub.uni-koeln.de/id/eprint/4231
