# Supplementary material for: Large scale and regional demographic responses to climatic changes in Europe during the Final Palaeolithic
Source: PLoS One. 2025 Apr 2;20(4):e0310942. doi: 10.1371/journal.pone.0310942 (PMC11964466; doi:10.1371/journal.pone.0310942)
Supplement: S2 Table — The table comprises only data that were considered for calculation in this study (n = 39). Below each region, the corresponding quartiles (Q1, Q2, Q3) used to calculate the palaeodemographic estimates are shown (see table 2). (DOCX) [file pone.0310942.s003.docx]

**S2 Table.** **Sites with data on Raw Material Catchment Areas (RMCA) assigned to GS-1.**

| **Region** | **Site** | **RMCA in km^2^** |  | **Reference** |
| --- | --- | --- | --- | --- |
| NW |  |  |  |  |
|  | Geldrop 3-1 | 797 |  | [1] |
|  | Altenrath-Ziegenberg | 3646 |  | [2]; pers. comm. B. Gehlen |
|  | Reingsen I | 6728 |  | [3–5]; pers. comm. B. Gehlen |
|  | Kartstein | 9665 |  | [6]; pers. comm. B. Gehlen |
| *NW* | *Q1* | *2934* |  |  |
|  | *Q2* | *5187* |  |  |
|  | *Q3* | *7462* |  |  |
| NE |  |  |  |  |
|  | Całowanie | 605 |  | [7] |
|  | Chełmno 4 (Chelmno) | 963 |  | [8] |
|  | Rzuchów 24 | 963 |  | [8] |
|  | Janów 21 | 976 |  | [8] |
|  | Dzierżysław (Dzierzyslaw) | 1302 |  | [9] |
|  | Gönnersdorf | 4959 |  | [10] |
|  | Cichmiana 2 | 7924 |  | [8] |
|  | Fürth-Atzenhof-NO | 11,408 |  | [11] |
|  | Blanice 6 | 18,244 |  | [12] |
|  | Uckersdorf | 24,016 |  | [11] |
|  | Wurz "Auf der Schlattein" | 24,813 |  | [11] |
| *NE* | *Q1* | *970* |  |  |
|  | *Q2* | *4959* |  |  |
|  | *Q3* | *14,826* |  |  |
| SE |  |  |  |  |
|  | Sattenbeuren-Kieswerk | 876 |  | [13] |
|  | Abri Wachtfels | 2529 |  | [14] |
|  | Abri Unter den Seewänden | 2857 |  | [15] pers. comm. B. Gehlen |
|  | "Gemeindebeunden" Bad Buchau-Kappel | 3056 |  | [16] |
|  | Geispel | 3474 |  | [14] |
|  | Lengnau-Chlini Ey | 3781 |  | [14]; pers. comm. J. Affolter / B. Gehlen |
|  | Fru, La (423) | 3864 |  | [17]; pers. comm. J. Affolter / B. Gehlen |
|  | Feuerbichl - Schwangau-Horn | 4807 |  | [18,19]; pers. comm. B. Gehlen |
|  | Lüscherzmoos | 8297 |  | [14]; pers. comm. J. Affolter / B. Gehlen |
|  | Cham-Grindel III | 9026 |  | [14] |
|  | Langrüti | 10,309 |  | [14] |
|  | Altwasser-Höhle 1 | 10,943 |  | [14] |
|  | Fürsteiner | 15,239 |  | [14] |
|  | Cham-Grindel I | 18,836 |  | [14] |
| *SE* | *Q1* | *3160* |  |  |
|  | *Q2* | *4336* |  |  |
|  | *Q3* | *9988* |  |  |
| SW |  |  |  |  |
|  | Champ Chalatras | 1524 |  | [20] |
|  | Borie del Rey (426), La | 1982 |  | [21] |
|  | Balzola, Cueva de | 2401 |  | [22] |
|  | Urratxa III | 2479 |  | [23] |
|  | Troubat, La Grotte-Abri de/ Moulin, La grott | 2522 |  | [24] |
|  | Cuze de sainte-Anastasie UA4 | 6153 |  | [25] |
|  | Peyrazet, Le Grotte Abri de | 7172 |  | [26,27] |
|  | Port-de-Penne | 15,068 |  | [21] |
|  | Mas d'Azil | 40,446 |  | [28] |
|  | Cuze de sainte-Anastasie UA5 | 49,828 |  | [25] |
| *SW* | *Q1* | *2420* |  |  |
|  | *Q2* | *4337* |  |  |
|  | *Q3* | *13094* |  |  |
|  |  |  |  |  |

The table comprises only data that were considered for calculation in this study (n = 39). Below each region, the corresponding quartiles (Q1, Q2, Q3) used to calculate the palaeodemographic estimates are shown (see **table 2**).

**References**

1. Deeben J. De laatpaleolithische en mesolithische sites bij Geldrop (N. Br.). Deel 5. Archeologie. 1999;9: 3–35.

2. Floss H. Rohmaterialversorgung im Paläolithikum des Mittelrheingebietes. Bonn: Habelt; 1994.

3. Blank R. Ein Fundplatz der endpaläolithischen Stielspitzen - Gruppe am nördlichen Mittelgebirgsrand. Arch Korr. 1985;15: 287–292.

4. Balthasar P. Untersuchung des Wandels der Steinartefaktgrundproduktion in der Westfälischen Bucht vom Spätpaläolithikum bis zum Mesolithikum. Ph.D., Friedrich-Schiller-Universität. 2019.

5. Blank R. Reingsen I, Stadt Iserlohn, Märkischer Kreis. In: Günther K, editor. Alt- und mittelsteinzeitliche Fundplätze in Westfalen Teil 2: Altsteinzeitliche Fundplätze in Westfalen. Münster: Westfäl. Museum für Archäologie; 1988. pp. 148–150.

6. Baales M. Umwelt und Jagdökonomie der Ahrensburger Rentierjäger im Mittelgebirge. Bonn: Dr. Rudolf Habelt GmbH; 1996.

7. Sulgostowska Z. Final Palaeolithic Societies’ Mobility in Poland as Seen from the Distribution of Flints. Arch Baltica. 2006;7: 36–42.

8. Płaza DK, Kittel P, Petera-Zganiacz J, Dzieduszyńska DA, Twardy J. Late Palaeolithic settlement pattern in palaeogeographical context of the river valleys in the Koło Basin (Central Poland). Quat Int. 2015;370: 40–54. doi:10.1016/j.quaint.2014.09.058

9. Tarbska J, Walanus A, Ciesielczuk J, Samek L, Dutkiewicz E. Ferruginous Raw Material Sources for Palaeolithic in Poland (Central Europe)? Provenance Studies: Occurrence, Litostratigraphy and Application. Jerusalem; 2008. Available: https://www.ndt.net/?id=6175

10. Baales M. Archäologie des Eiszeitalters: frühe Menschen an Mittelrhein und Mosel. Koblenz: Gesellschaft für Archäologie an Mittelrhein und Mosel; 2005.

11. Sauer F. Late Palaeolithic Land Use Patterns in Bavaria. Ph.D., Friedrich-Alexander-Universität. 2018. Available: urn:nbn:de:bvb:29-opus4-92875

12. Vencl S. Prehistory of Bohemia 1: The Palaeolithic and Mesolithic. Praha: Archeologický ústav AV CR; 2013.

13. Kind C-J. Sattenbeuren - Kieswerk, ein spätpaläolithischer Uferrandlagerplatz am Federsee. Fundb Baden-Württemberg. 1995;20: 159–194.

14. Affolter J. Silexrohstoffe - Schlüssel zu Analyse von Beziehungsnetzen. Die letzten Wildbeuter der Eiszeit - Neue Forschungen zum Spätpaläolithikum im Kanton Basel-Landschaft. Bern: Schwabe Verlag; 2015. pp. 198–209.

15. Gehlen B. Rast am Fuße der Alpen: Die allerødzeitliche Abristation “Unter den Seewänden” bei Füssen im Ostallgäu. Zeit-Räume: Gedenkschrift für Wolfgang Taute. Propylaeum; 2001. pp. 475–552. doi:10.11588/PROPYLAEUM.245.327

16. Jochim MA, Kind C-J, Kleinmann A, Merkt J, Stephan E. Eine spätpaläolithische Fundstelle am Ufer des Federsees: Bad Buchau-Kappel, Flurstück Gemeindebeunden. Fundber Baden-Württemberg. 2015;35: 37–134. doi:10.11588/FBBW.2015.0.44521

17. Mevel L, Fornage-Bontemps C, Bereiziat G. Au carrefour des influences culturelles ? Les industries lithiques de la fin du Tardiglaciaire entre Alpes du nord et Jura, 11 500-9 500 CalBC. In: Langlais M, Naudinot N, Peresani M, editors. Les groupes culturels de la transition Pléistocène - Holocène entre Atlantique et Adriatique: actes de la séance de la Société préhistorique française Bordeaux 24-25 mai 2012. Paris: Société Préhistorique Française; 2014. pp. 45–81.

18. Gehlen B. Steinzeitliche Funde im östlichen Allgäu. In: Küster H, editor. Vom Werden einer Kulturlandschaft Vegetationsgeschichtliche Studien am Auerberg (Südbayern). Weinheim; 1988. pp. 195–209.

19. Gehlen B. Schwangau, Lkr. Ostallgäu. Spätmesolithische Freilandstationen im Forggensee. In: Czysz W, Dietrich H, Weber G, editors. Kempten und das Allgäu Führer zu archäologischen Denkmälern in Deutschland. Stuttgart: Theiss; 1995. pp. 223–224.

20. Pasty J-F, Alix Ph, Ballut C, Umr, Clermont-Ferrand RL, Griggo C, et al. Le gisement épipaléolithique à pointes de Malaurie de Champ-Chalatras (Les Martres d’Artière, Puy-de-Dôme). Paleo. 2002; 101–176. doi:10.4000/paleo.1540

21. Langlais M, Detrain L, Ferrié J-G, Mallye J-B, Marquebielle B, Rigaud S, et al. Réévaluation des gisements de La Borie del Rey et de Port-de-Penne: Nouvelles perspectives pour la transition Pléistocène-Holocène dans le Sud-Ouest de la France. In: Langlais M, Naudinot N, Peresani M, editors. Les groupes culturels de la transition Pléistocène - Holocène entre Atlantique et Adriatique: Actes de la Séance de la Société Préhistorique Française: Bordeaux: 24-25 Mai 2012. Paris: Société Préhistorique Française; 2014. pp. 83–128.

22. García Rojas M. Dinámicas de talla y gestión de las materias primas silíceas a finales del pleistoceno en el País Vasco. Ph.D., Universidad del País Vasco. 2014. Available: http://hdl.handle.net/10810/18142

23. Berganza Gochi E. El tránsito del Tardiglacial al Holoceno en el País Vasco. Munibe Antropologia - Arkeologia. 2005;57: 249–258.

24. Barbaza M. Environmental changes and cultural dynamics along the northern slope of the Pyrenees during the Younger Dryas. Quat Int. 2011;242: 313–327. doi:10.1016/j.quaint.2011.03.012

25. Langlais M, Delvigne V, Gibaud A, Jacquier J, Perrin T, Fernandes P, et al. La séquence archéostratigraphique du Cuze de Sainte-Anastasie (Cantal) : variations diachroniques et synchroniques des industries lithiques du Laborien au Mésolithique. Bull Soc Préhist Fr. 2018;115: 497–529. doi:10.3406/bspf.2018.14921

26. Langlais M, Laroulandie V, Jacquier J, Costamagno S, Chalard P, Mallye J-B, et al. Le Laborien récent de la grotte-abri de Peyrazet (Creysse, Lot, France). Nouvelles données pour la fin du Tardiglaciaire en Quercy. Paleo. 2015; 79–116. doi:10.4000/paleo.2917

27. Langlais M, Laroulandie V, Bruxelles L, Chalard P, Cochard D, Costamagno S, et al. Les fouilles de la grotte-abri de Peyrazet (Creysse, Lot) : nouvelles données pour le Tardiglaciaire quercinois. Bull Soc Préhist Fr. 2009;106: 150–152. doi:10.3406/bspf.2009.13836

28. Kegler JF. Das Azilien von Mas d’Azil. Der chronologische und kulturelle Kontext der Rückenspitzengruppen in Südwesteuropa. Mit einem Beitrag von Jan F. Kegler und Stefan R. Loew. Ph.D., Universität zu Köln. 2007. Available: http://kups.ub.uni-koeln.de/id/eprint/4231
