# Supplementary material for: Large scale and regional demographic responses to climatic changes in Europe during the Final Palaeolithic
Source: PLoS One. 2025 Apr 2;20(4):e0310942. doi: 10.1371/journal.pone.0310942 (PMC11964466; doi:10.1371/journal.pone.0310942)
Supplement: S3 Table — Thin horizontal lines indicate regions with transfer of RMCA data (cf. S3 Fig). ODI = optimally describing Isoline in km²; Q = Quartile [1 = lower, 2 = median, 3 = upper]; RMCA (km²) = raw material catchment area in km²; Nraw = number of RMCAs; Ngroups = number of groups; Npersons = number of persons; Dpopulation = population density within Core Areas as p/km2, and the Total Area of Calculation (bottom rows). (DOCX) [file pone.0310942.s004.docx]

**S3 Table.** **Regionally distinguished demographic estimates for GI-1d-a based on dataset B (i.e., only directly dated or specifically attributable to GI-1d-a).**

| **Core Area region** | **ODI (km²)** | **Q** | **RMCA (km²)** | **N_raw_** | **N_groups_** | **N_persons_** | **D_population_** |
| --- | --- | --- | --- | --- | --- | --- | --- |
|  |  | 1 | 2670 |  | 18.3 | 777 | 0.016 |
| Great Britain | 48,810 | 2 | 5117 | 17 | **9.5** | **405** | **0.008** |
|  |  | 3 | 5896 |  | 8.3 | 352 | 0.007 |
|  |  | 1 |  |  | 10.3 | 436 | 0.016 |
| N France | 27,421 | 2 |  |  | **5.4** | **228** | **0.008** |
|  |  | 3 |  |  | 4.7 | 198 | 0.007 |
|  |  | 1 |  |  | 36.9 | 1567 | 0.016 |
| Benelux & NW Germany | 98,467 | 2 |  |  | **19.2** | **818** | **0.008** |
|  |  | 3 |  |  | 16.7 | 710 | 0.007 |
|  |  | 1 |  |  | 7.3 | 308 | 0.016 |
| S Scandinavia | 19,372 | 2 |  |  | **3.8** | **161** | **0.008** |
|  |  | 3 |  |  | 3.3 | 140 | 0.007 |
|  |  | 1 |  |  | 34.7 | 1473 | 0.016 |
| Poland & NE Germany | 92,553 | 2 |  |  | **18.1** | **769** | **0.008** |
|  |  | 3 |  |  | 15.7 | 667 | 0.007 |
|  |  | 1 | 1057 |  | 34.2 | 1455 | 0.040 |
| Czech Rep. & SE Germany | 36,181 | 2 | 1948 | 10 | **18.6** | **789** | **0.022** |
|  |  | 3 | 2626 |  | 13.8 | 585 | 0.016 |
|  |  | 1 | 2604 |  | 7.6 | 323 | 0.016 |
| Switzerland & SW Germany | 19,783 | 2 | 3550 | 19 | **5.6** | **237** | **0.012** |
|  |  | 3 | 8662 |  | 2.3 | 97 | 0.005 |
|  |  | 1 |  |  | 5.3 | 224 | 0.016 |
| Italy | 13,745 | 2 |  |  | **3.9** | **165** | **0.012** |
|  |  | 3 |  |  | 1.6 | 67 | 0.005 |
|  |  | 1 |  |  | 7.9 | 334 | 0.016 |
| SE France | 20,470 | 2 |  |  | **5.8** | **245** | **0.012** |
|  |  | 3 |  |  | 2.4 | 100 | 0.005 |
|  |  | 1 | 1578 |  | 23.4 | 996 | 0.027 |
| SW France | 36,993 | 2 | 2522 | 7 | **14.7** | **624** | **0.017** |
|  |  | 3 | 20,730 |  | 1.8 | 76 | 0.002 |
|  |  | 1 |  |  | 41.6 | 1768 | 0.027 |
| Spain & French Pyrenees | 65,661 | 2 |  |  | **26.0** | **1107** | **0.017** |
|  |  | 3 |  |  | 3.2 | 135 | 0.002 |
|  |  | 1 |  |  | 6.1 | 261 | 0.027 |
| Portugal | 9700 | 2 |  |  | **3.8** | **163** | **0.017** |
|  |  | 3 |  |  | 0.5 | 20 | 0.002 |
|  |  | 1 |  |  | ***234*** | ***9924*** | ***0.020*** |
| **Sum Core Area** | ***489,156*** | 2 |  | **53** | **134** | **5710** | **0.012** |
|  |  | 3 |  |  | ***74*** | ***3147*** | ***0.006*** |
|  |  | 1 |  |  |  |  | ***0.004*** |
| **Total Area of Calculation** | ***2,600,000*** | 2 |  |  |  |  | **0.002** |
|  |  | 3 |  |  |  |  | ***0.001*** |

Thin horizontal lines indicate regions with transfer of RMCA data (cf. **S3 Fig**). ODI = optimally describing Isoline in km²; Q = Quartile [1 = lower, 2 = median, 3 = upper]; RMCA (km²) = raw material catchment area in km²; N_raw_ = number of RMCAs; N_groups_ = number of groups; N_persons_ = number of persons; D_population_ = population density within Core Areas as p/km^2^, and the Total Area of Calculation (bottom rows).
