# Supplementary material for: Large scale and regional demographic responses to climatic changes in Europe during the Final Palaeolithic
Source: PLoS One. 2025 Apr 2;20(4):e0310942. doi: 10.1371/journal.pone.0310942 (PMC11964466; doi:10.1371/journal.pone.0310942)
Supplement: S4 Table — Thin horizontal lines indicate regions with transfer of RMCA data (cf. S4 Fig). For explanation on abbreviations see S2 Table. (DOCX) [file pone.0310942.s005.docx]

**S4 Table.** **Regionally distinguished demographic estimates for GS-1 based on dataset B (i.e., only directly dated or specifically attributable to GS-1).**

| **Core Area region** | **ODI (km²)** | | **Q** | | **RMCA (km²)** | | **N_raw_** | | **N_groups_** | | **N_persons_** | | **D_population_** | |
| --- | --- | --- | --- | --- | --- | --- | --- | --- | --- | --- | --- | --- | --- | --- |
|  |  | | 1 | | 2934 | |  | | *8.4* | | *358* | | *0.014* | |
| Great Britain | 24,720 | | 2 | | 5187 | | 4 | | **4.8** | | **203** | | **0.008** | |
|  |  | | 3 | | 7462 | |  | | *3.3* | | *141* | | *0.006* | |
|  |  | | 1 | |  | |  | | *1.1* | | *49* | | *0.014* | |
| N France | 3360 | | 2 | |  | |  | | **0.6** | | **28** | | **0.008** | |
|  |  | | 3 | |  | |  | | *0.5* | | *19* | | *0.006* | |
|  |  | | 1 | |  | |  | | *17.4* | | *740* | | *0.014* | |
| Benelux & NW Germany | 51,060 | | 2 | |  | |  | | **9.8** | | **418** | | **0.008** | |
|  |  | | 3 | |  | |  | | *6.8* | | *291* | | *0.006* | |
|  |  | | 1 | |  | |  | | *0.4* | | *18* | | *0.014* | |
| S Scandinavia | 1220 | | 2 | |  | |  | | **0.2** | | **10** | | **0.008** | |
|  |  | | 3 | |  | |  | | *0.2* | | *7* | | *0.006* | |
|  |  | | 1 | |  | |  | | *49.0* | | *2085* | | *0.014* | |
| Poland & NE Germany | 143,890 | | 2 | |  | |  | | **27.7** | | **1179** | | **0.008** | |
|  |  | | 3 | |  | |  | | *19.3* | | *819* | | *0.006* | |
|  |  | | 1 | | 970 | |  | | *10.1* | | *429* | | *0.044* | |
| Czech Rep. & SE Germany | 9800 | | 2 | | 4959 | | 11 | | **2.0** | | **84** | | **0.009** | |
|  |  | | 3 | | 14,826 | |  | | *0.7* | | *28* | | *0.003* | |
|  |  | | 1 | | 3160 | |  | | *8.5* | | *361* | | *0.013* | |
| Switzerland & SW Germany | 26,880 | | 2 | | 4336 | | 14 | | **6.2** | | **263** | | **0.010** | |
|  |  | | 3 | | 9988 | |  | | *2.7* | | *114* | | *0.004* | |
|  |  | | 1 | |  | |  | | *9,5* | | *405* | | *0,013* | |
| Italy | 30,120 | | 2 | |  | |  | | **6,9** | | **295** | | **0,010** | |
|  |  | | 3 | |  | |  | | *3,0* | | *128* | | *0,004* | |
|  |  | | 1 | |  | |  | | *3,2* | | *136* | | *0,013* | |
| SE France | 10090 | | 2 | |  | |  | | **2,3** | | **99** | | **0,010** | |
|  |  | | 3 | |  | |  | | *1,0* | | *43* | | *0,004* | |
|  |  | | 1 | | 2420 | |  | | *9,4* | | *401* | | *0,018* | |
| SW France | 22,860 | | 2 | | 4337 | | 10 | | **5,3** | | **224** | | **0,010** | |
|  |  | | 3 | | 13,094 | |  | | *1,7* | | *74* | | *0,003* | |
|  |  | | 1 | |  | |  | | *25,0* | | *1065* | | *0,018* | |
| Spain & French Pyrenees | 60,630 | | 2 | |  | |  | | **14,0** | | **594** | | **0,010** | |
|  |  | | 3 | |  | |  | | *4,6* | | *197* | | *0,003* | |
|  |  | | 1 | |  | |  | | *3.1* | | *133* | | *0.018* | |
| Portugal | 7560 | | 2 | |  | |  | | **1.7** | | **74** | | **0.010** | |
|  |  | | 3 | |  | |  | | *0.6* | | *25* | | *0.003* | |
|  |  | | 1 | |  | |  | | ***145*** | | ***6179*** | | ***0,016*** | |
| **Sum Core Area** | ***392,190*** | | 2 | |  | | **39** | | **82** | | **3471** | | **0,009** | |
|  |  | | 3 | |  | |  | | ***44*** | | ***1886*** | | ***0,005*** | |
|  | |  | | 1 | |  | |  | |  | |  | | ***0.002*** |
| **Total Area of Calculation** | | ***2,600,000*** | | 2 | |  | |  | |  | |  | | **0.001** |
|  | |  | | 3 | |  | |  | |  | |  | | ***0.001*** |

Thin horizontal lines indicate regions with transfer of RMCA data (cf. **S4 Fig**). For explanation on abbreviations see **S2** **Table**.
