# Supplementary material for: Large scale and regional demographic responses to climatic changes in Europe during the Final Palaeolithic
Source: PLoS One. 2025 Apr 2;20(4):e0310942. doi: 10.1371/journal.pone.0310942 (PMC11964466; doi:10.1371/journal.pone.0310942)
Supplement: S5 Table — The numbers are given for the entire database (“all radiocarbon dates”, upper), for the Total Area of Calculation (middle) and for each region (lower). Dates were taken from the Radiocarbon Palaeolithic Europe Database v.27 [49], see details in main publication. Except for the first dataset (n = 1758), radiocarbon dates per site and time-bin were counted as one occurrence each (n = 731). To account for the slightly shorter phase of the GS-1 (1.2 ky) compared to GI-1d-a (1.3 ky), the GS-1 radiocarbon counts were normalised for the duration. Note that the regions slightly differ from the ones defined in Fig 1 (that is for “Benelux and W Germany” and Switzerland). (DOCX) [file pone.0310942.s006.docx]

**S5 Table.** **Number of radiocarbon-dated sites divided by GI-1d-a and GS-1**.

|  | **period** | **ky (duration)** | **n of radiocarbon dates** | **%** | **n normalised to 1.3 ky** | **%** |
| --- | --- | --- | --- | --- | --- | --- |
|  |  |  |  |  |  |  |
| all radiocarbon dates | GS-1 | 1.2 | 522 | 30 | 566 | 31 |
|  | GI-1d-a | 1.3 | 1236 | 70 |  | 69 |
| **Sum** |  |  | **1758** |  |  |  |
|  |  |  |  |  |  |  |
| **region** | **period** | **years (duration)** | **presence of radiocarbon dates per site and time-bin** | **% per region** | **normalised to 1.3 ky** | **% per region** |
|  |  |  |  |  |  |  |
| Total Area of Calculation | GS-1 | 1.2 | 276 | 38 | 302 | 40 |
|  | GI-1d-a | 1.3 | 455 | 62 |  | 60 |
| **Sum** |  |  | **731** |  |  |  |

|  |  |  |  |  |  |  |
| --- | --- | --- | --- | --- | --- | --- |
| Great Britain | GS-1 |  | 29 | 45 | 31 | 47 |
|  | GI-1d-a |  | 36 | 55 |  | 53 |
| N France >47.4°N | GS-1 |  | 12 | 41 | 13 | 43 |
|  | GI-1d-a |  | 17 | 59 |  | 57 |
| Benelux & W Germany | GS-1 |  | 26 | 29 | 28 | 31 |
|  | GI-1d-a |  | 63 | 71 |  | 69 |
| S Scandinavia | GS-1 |  | 7 | 39 | 8 | 41 |
|  | GI-1d-a |  | 11 | 61 |  | 59 |
| Poland & NE Germany | GS-1 |  | 29 | 37 | 31 | 39 |
|  | GI-1d-a |  | 49 | 63 |  | 61 |
| Czech Rep. & SE Germany | GS-1 |  | 6 | 25 | 7 | 27 |
|  | GI-1d-a |  | 18 | 75 |  | 73 |
| Switzerland | GS-1 |  | 3 | 18 | 3 | 19 |
|  | GI-1d-a |  | 14 | 82 |  | 81 |
| Italy | GS-1 |  | 28 | 44 | 30 | 46 |
|  | GI-1d-a |  | 36 | 56 |  | 54 |
| SE France >1.8°E | GS-1 |  | 39 | 42 | 42 | 44 |
|  | GI-1d-a |  | 53 | 58 |  | 56 |
| SW France <1.8°E | GS-1 |  | 20 | 36 | 22 | 38 |
|  | GI-1d-a |  | 35 | 64 |  | 62 |
| Spain & French Pyrenees | GS-1 |  | 70 | 38 | 76 | 40 |
|  | GI-1d-a |  | 114 | 62 |  | 60 |
| Portugal | GS-1 |  | 7 | 44 | 8 | 46 |
|  | GI-1d-a |  | 9 | 56 |  | 54 |

The numbers are given for the entire database (“all radiocarbon dates”, upper), for the Total Area of Calculation (middle) and for each region (lower). Dates were taken from the Radiocarbon Palaeolithic Europe Database v.27 [1], see details in main publication. Except for the first dataset (n = 1758), radiocarbon dates per site and time-bin were counted as one occurrence each (n = 731). To account for the slightly shorter phase of the GS-1 (1.2 ky) compared to GI-1d-a (1.3 ky), the GS-1 radiocarbon counts were normalised for the duration. Note that the regions slightly differ from the ones defined in **Fig 1** (that is for “Benelux and W Germany” and Switzerland).

# **References**

1. Vermeersch PM. Radiocarbon Palaeolithic Europe database: A regularly updated dataset of the radiometric data regarding the Palaeolithic of Europe, Siberia included. Data in Brief. 2020;31: 105793. doi:10.1016/j.dib.2020.105793
