# Supplementary figures and images for: Large scale and regional demographic responses to climatic changes in Europe during the Final Palaeolithic
Source: PLoS One. 2025 Apr 2;20(4):e0310942. doi: 10.1371/journal.pone.0310942 (PMC11964466; doi:10.1371/journal.pone.0310942)

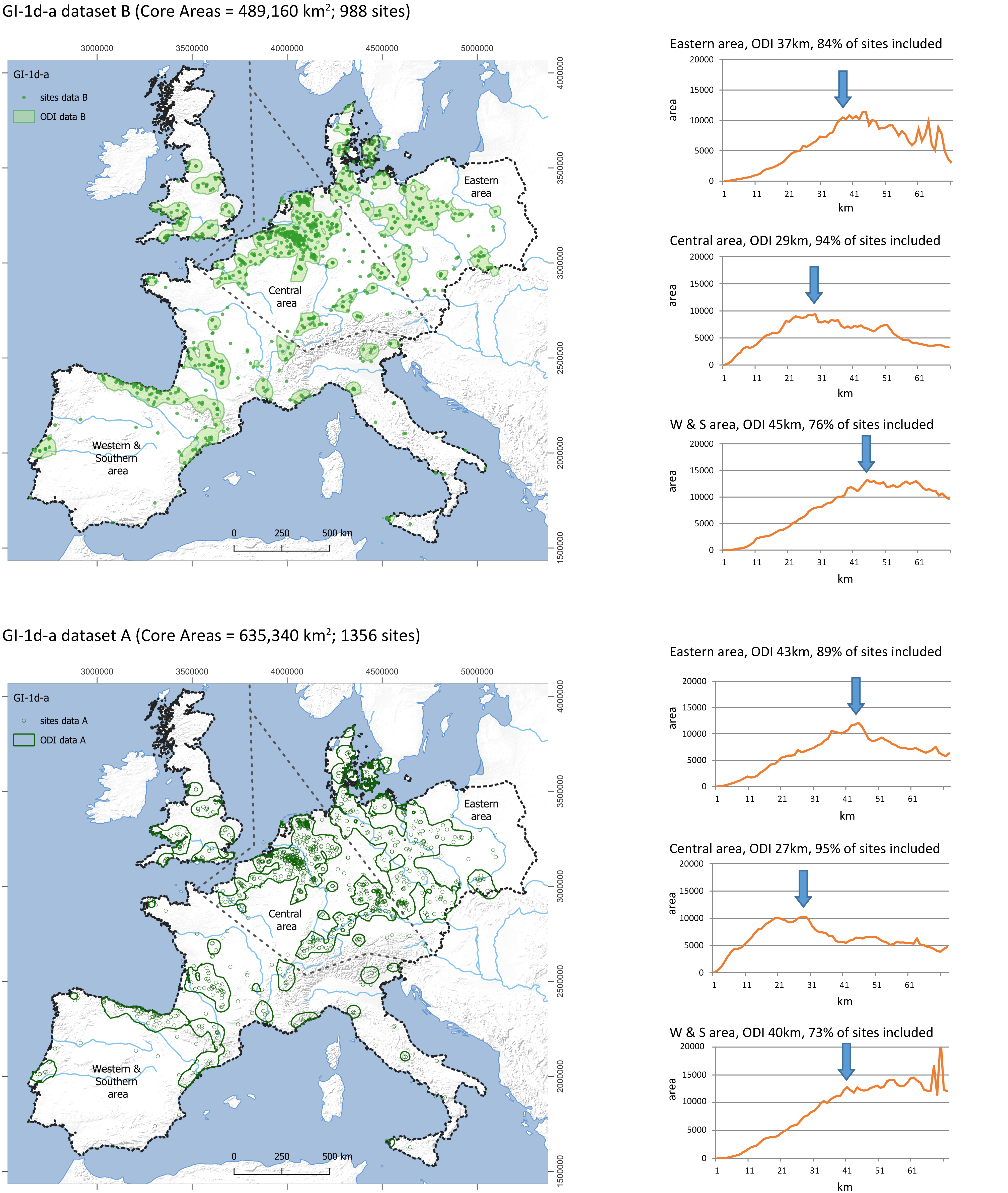

Supplement: S1 Fig — Regional areal increase of isolines (right) was interpolated by using distance values of Largest Empty Circles for GI-1d-a (right). X-axis of diagrams display radius of Largest Empty Circle, y-axis areal increase (km2) encircled by the Isoline. To define the Optimal Describing Isoline (ODI, arrows in diagrams), the first peak or plateau, encircling around 70% or more of all sites, is selected. Core Areas of dataset A comprises 90% of the sites, Core Areas of dataset B 85%. (TIF) [file pone.0310942.s007.tif]

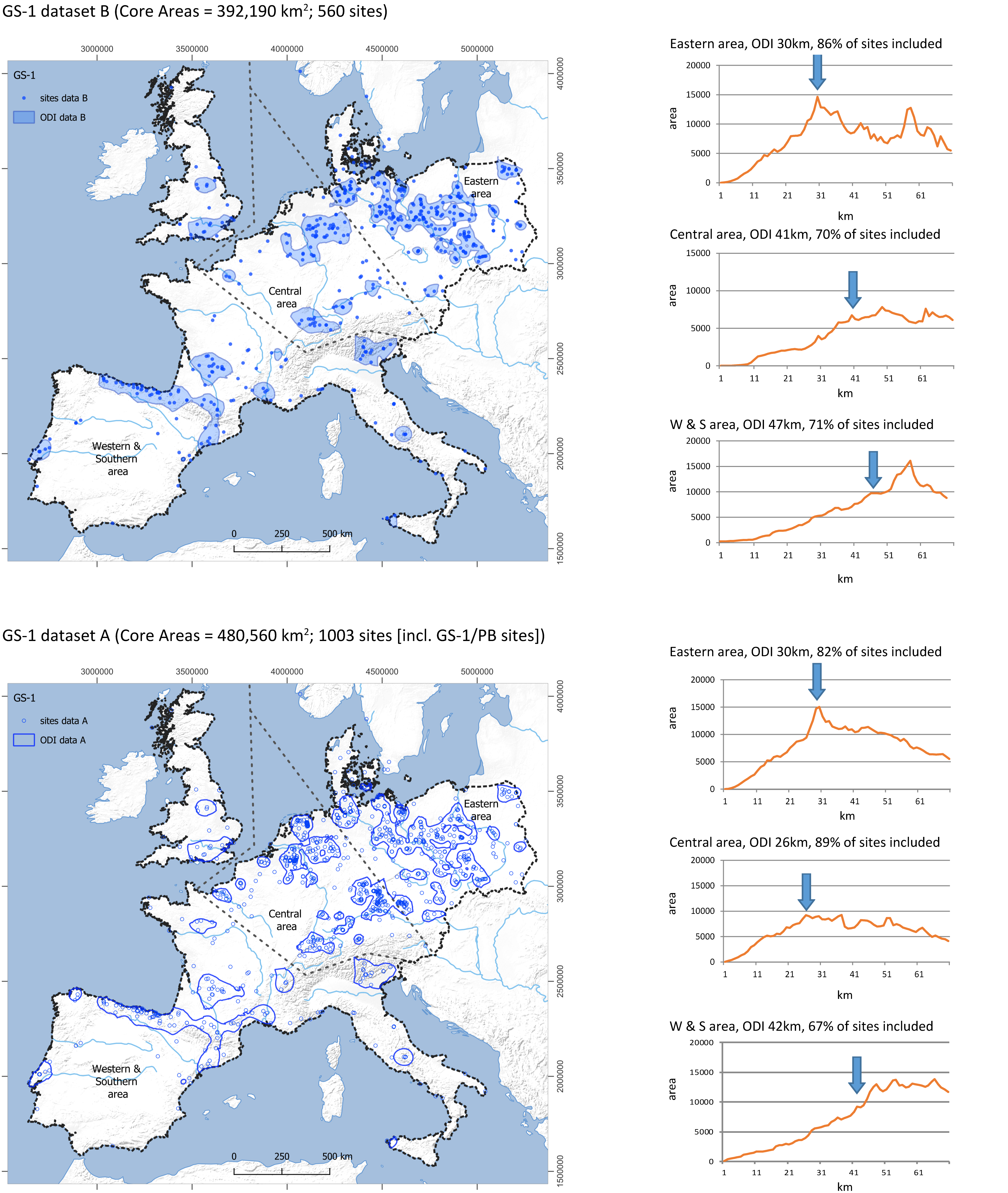

Supplement: S2 Fig — Dataset A comprises 82% of the sites, Core Areas of dataset B 74%. For explanation on details see S1 Fig. (TIF) [file pone.0310942.s008.tif]

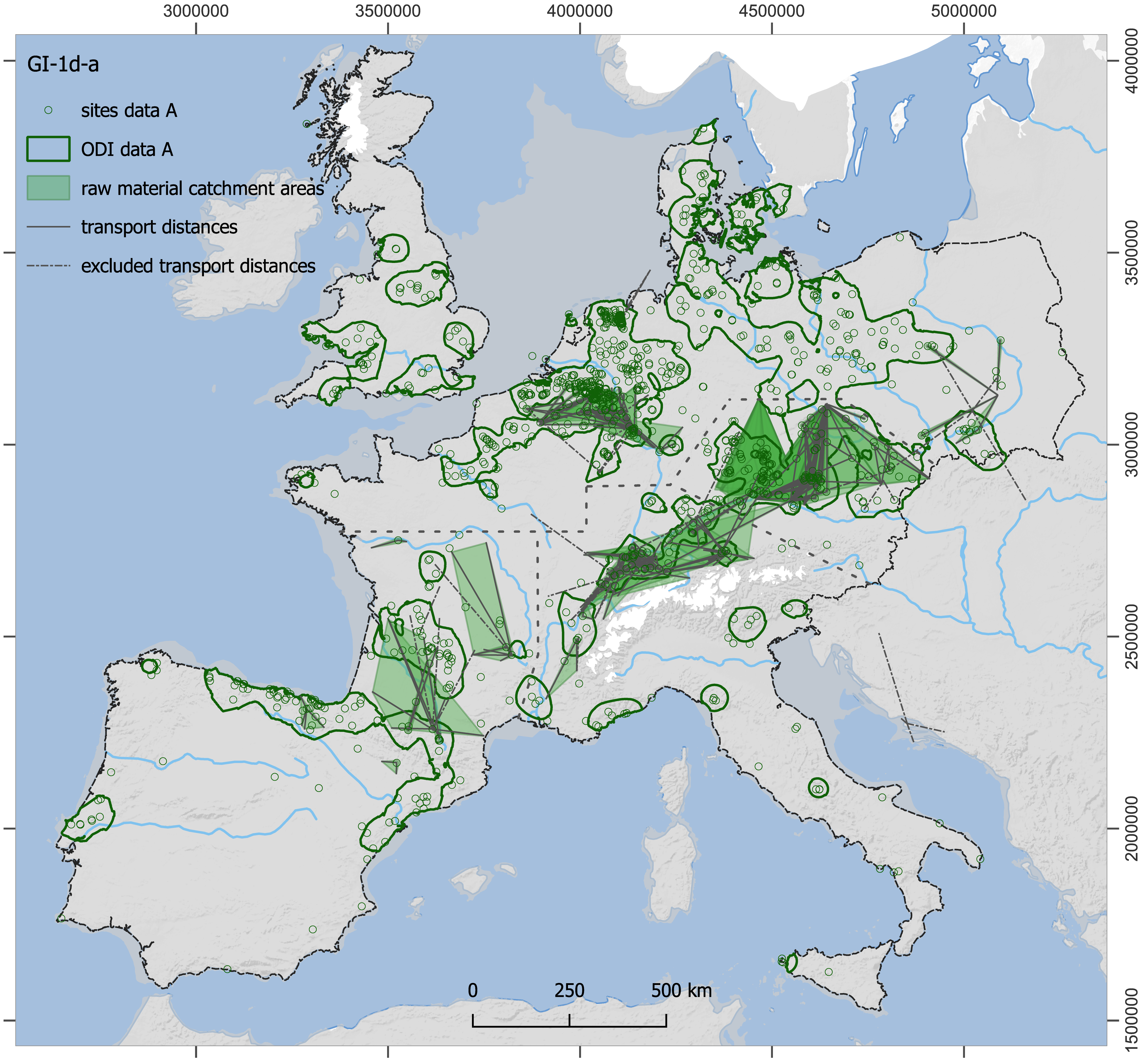

Supplement: S3 Fig — Thick dashed lines delimit regions within which the same RMCA data was used to calculate the demographic estimates. Background: Core Areas and sites from dataset A. (TIF) [file pone.0310942.s009.tif]

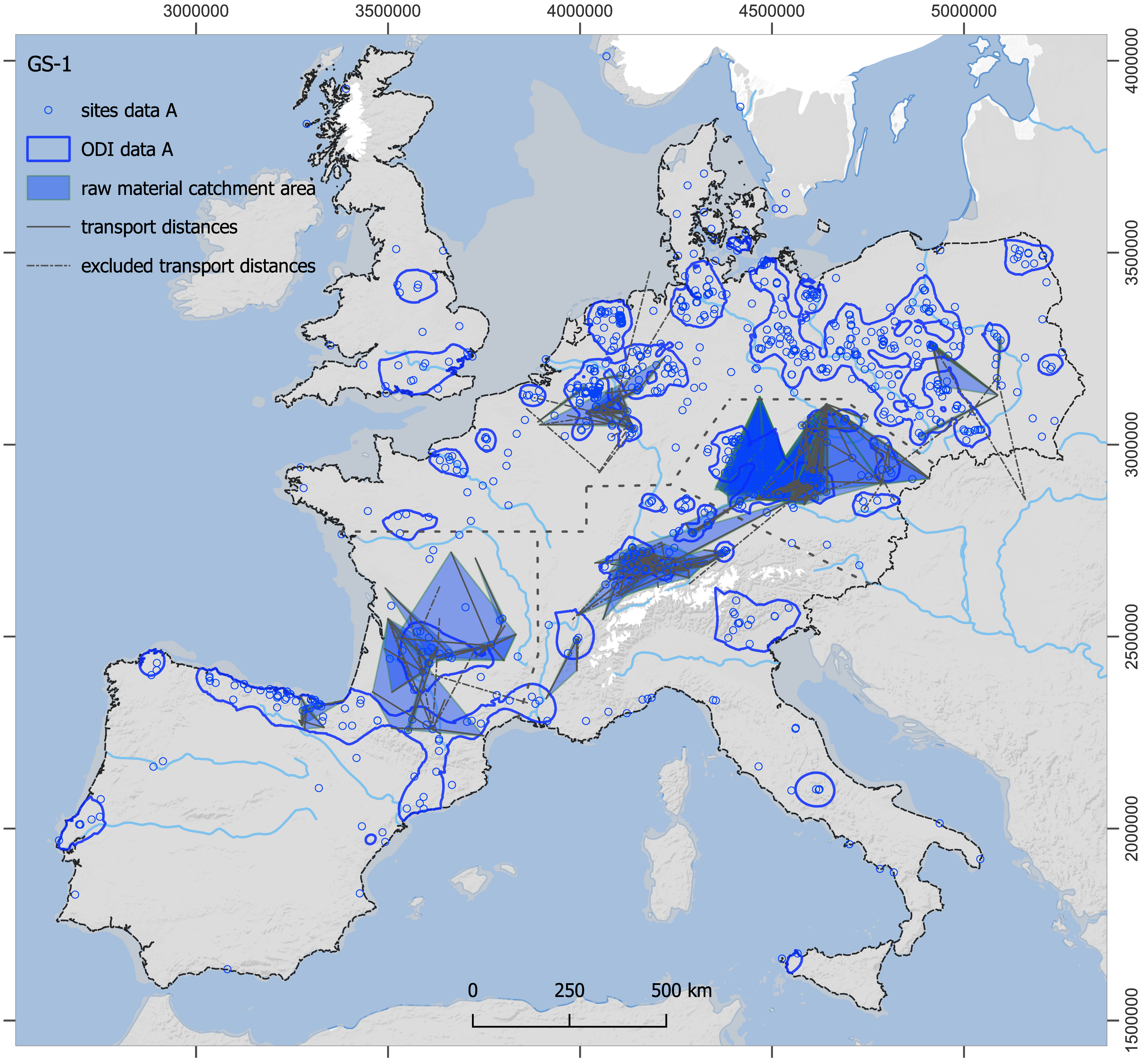

Supplement: S4 Fig — Thick dashed lines delimit regions within which the same RMCA data was used to calculate the demographic estimates. Background: Core Areas and sites from dataset A. (TIF) [file pone.0310942.s010.tif]

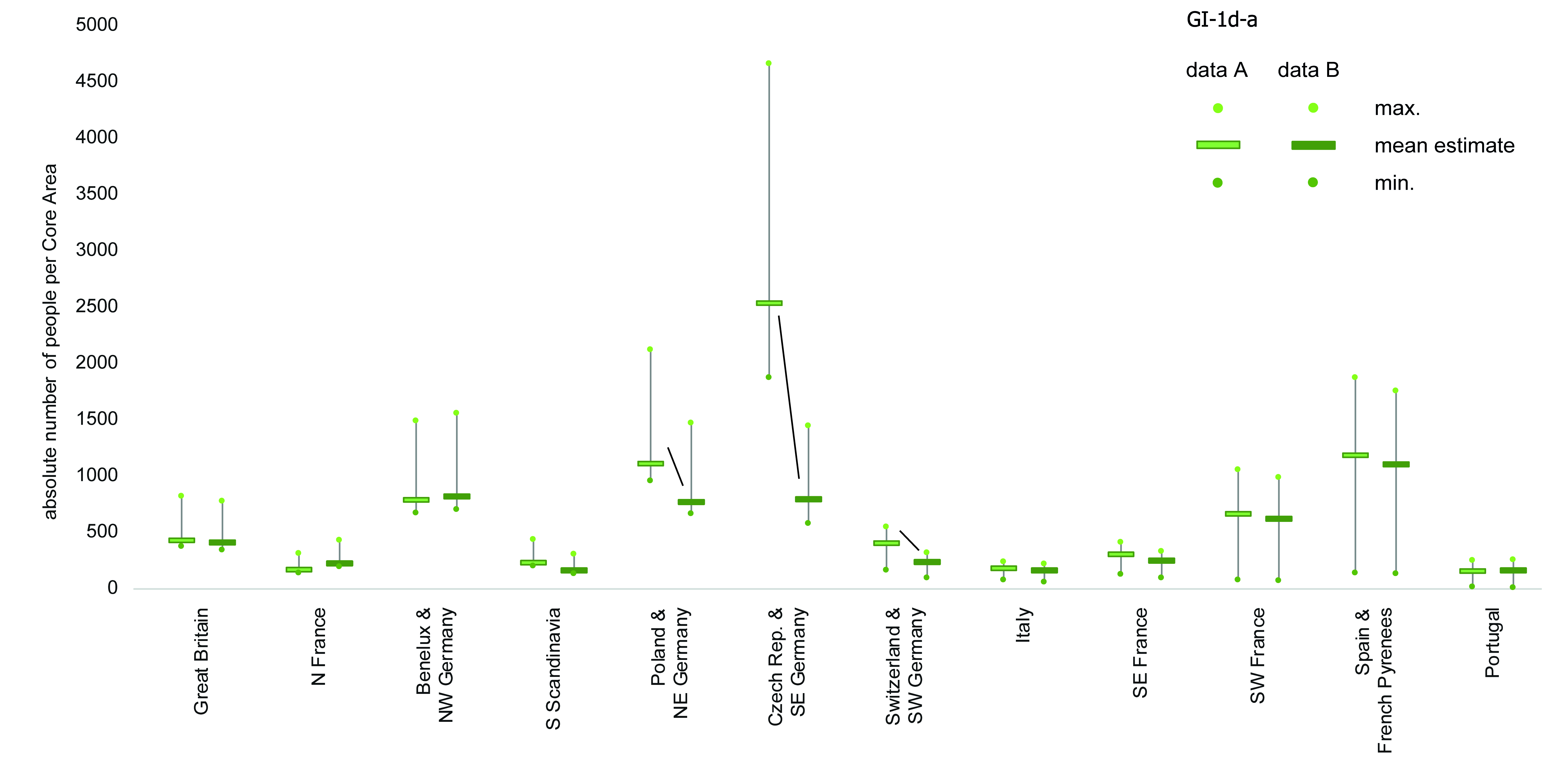

Supplement: S5 Fig — (TIF) [file pone.0310942.s011.tif]

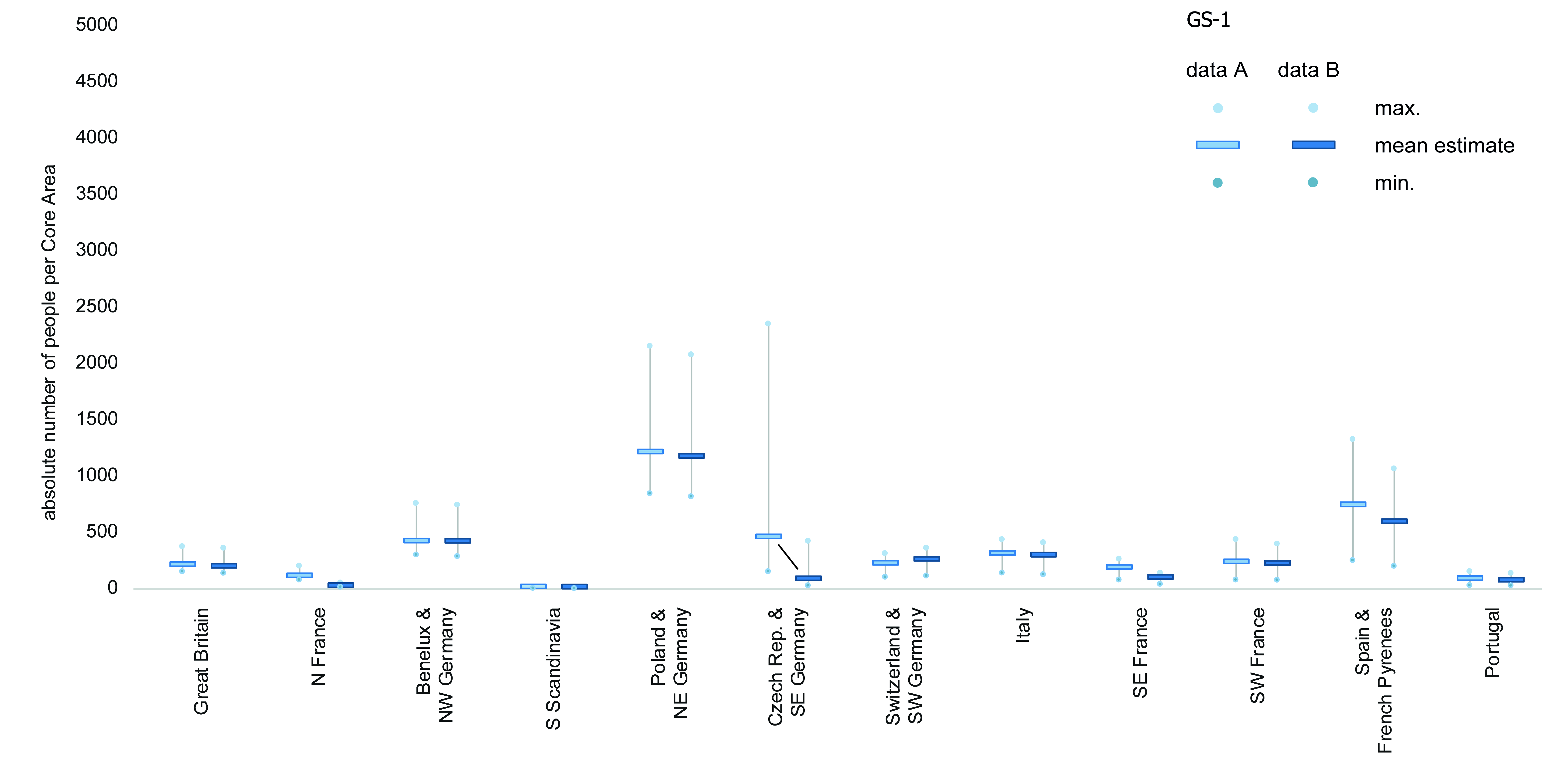

Supplement: S6 Fig — (TIF) [file pone.0310942.s012.tif]

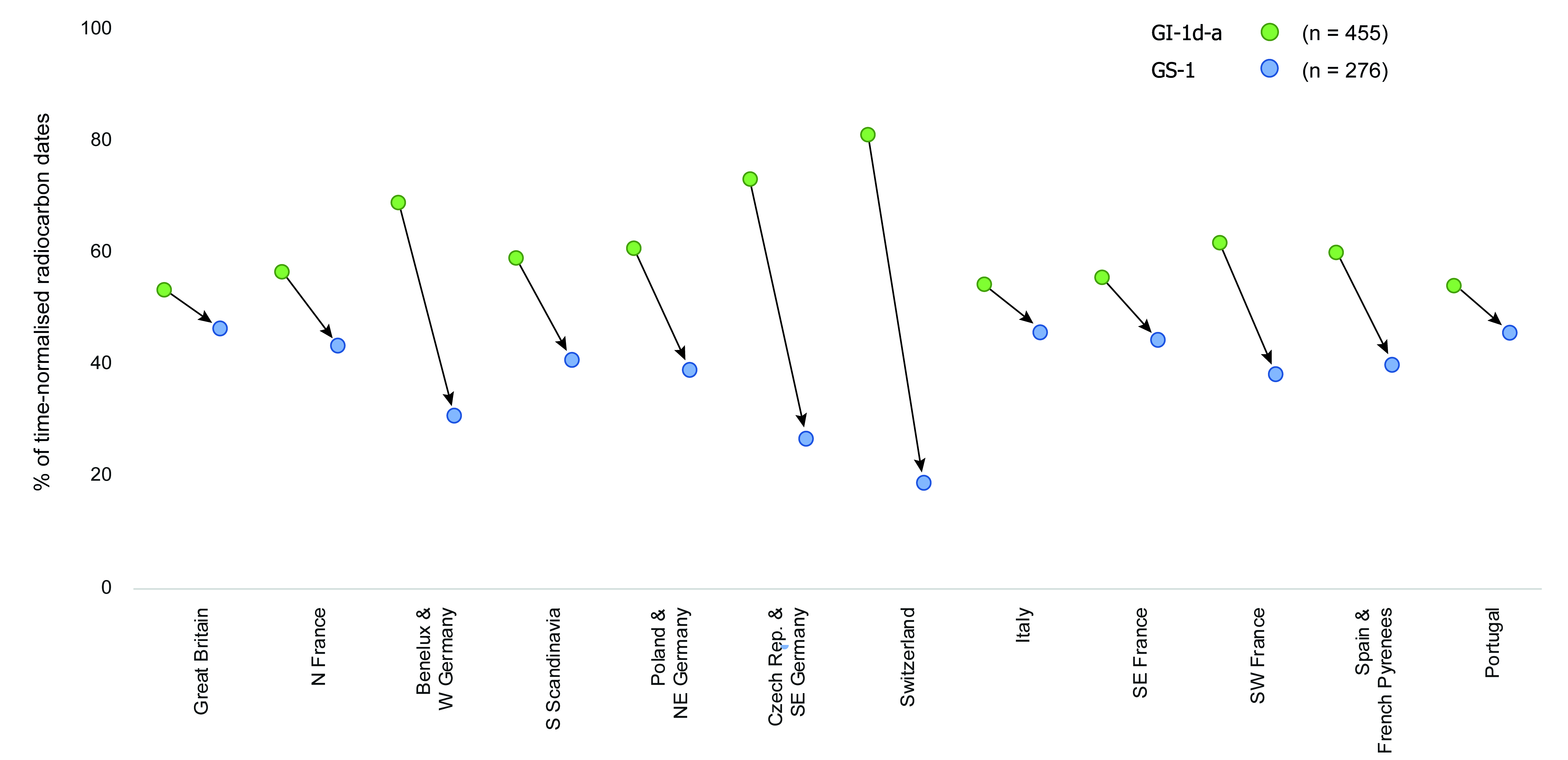

Supplement: S7 Fig — Dates were taken from the Radiocarbon Palaeolithic Europe Database v.27 [132]. We counted each time-bin covered by radiocarbon date(s) from an archaeological site as one occurrence (see S3 Table). To account for the slightly shorter phase of the GS-1 compared to GI-1d-a, the GS-1 radiocarbon counts were normalised for the duration of the phase. (TIF) [file pone.0310942.s013.tif]

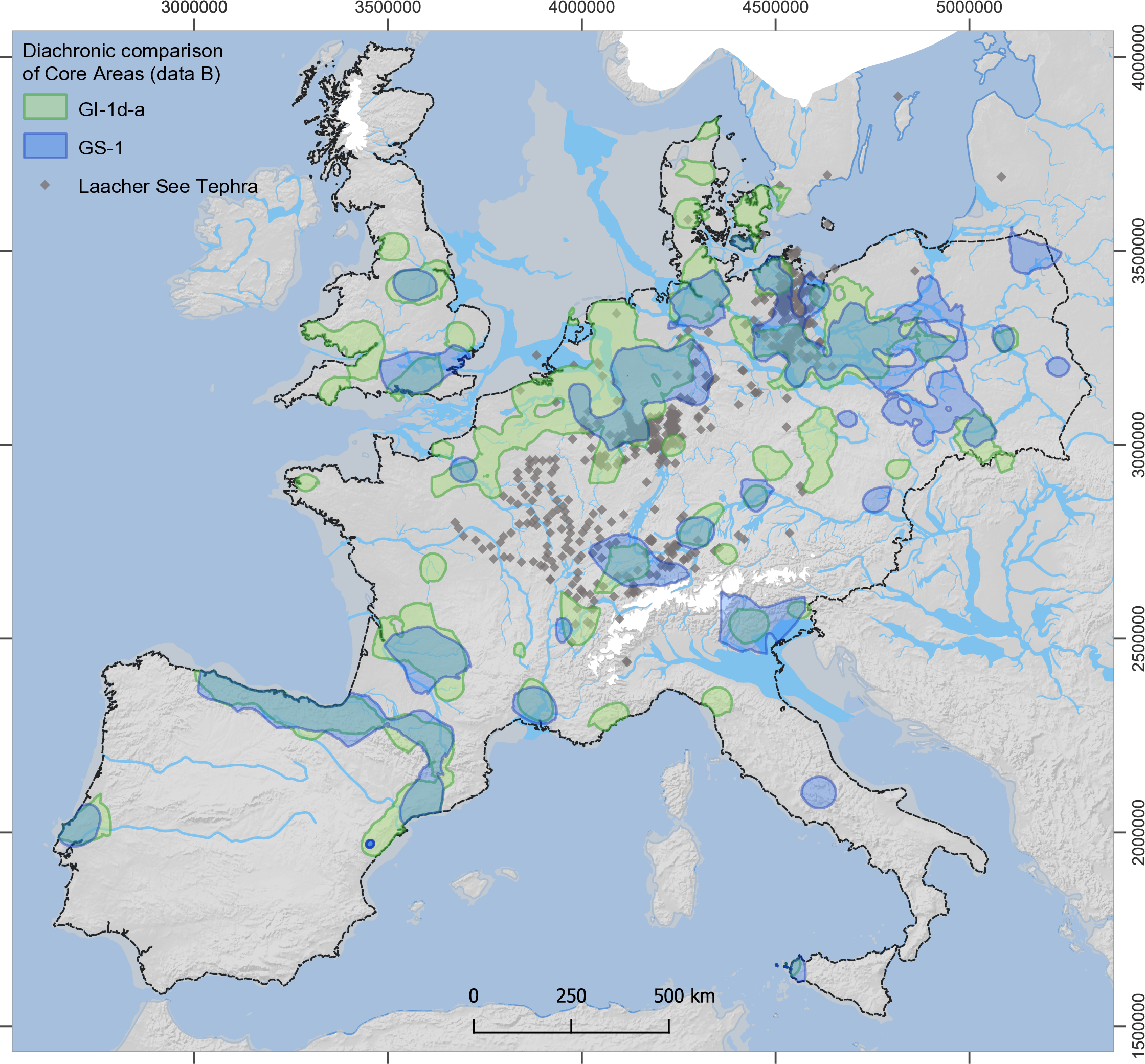

Supplement: S8 Fig — (TIF) [file pone.0310942.s014.tif]
